# The sim must go on: adapting resident education to the COVID-19 pandemic using telesimulation

**DOI:** 10.1186/s41077-020-00146-w

**Published:** 2020-09-29

**Authors:** Shivani M. Patel, Christina R. Miller, Adam Schiavi, Serkan Toy, Deborah A. Schwengel

**Affiliations:** Department of Anesthesiology and Critical Care Medicine, Johns Hopkins University, 1800 Orleans Street, Charlotte R. Bloomberg Children’s Center, Room 6349G, Baltimore, MD 21287 USA

**Keywords:** Simulation, Telesimulation, Education, Distance, Anesthesiologists, COVID-19, Faculty, Surveys and questionnaires, Pandemics, Feedback

## Abstract

The COVID-19 pandemic and social distancing rules necessitated the suspension of all in-person learning activities at our institution. Consequently, distance learning became essential. We adapted a high-fidelity immersive case-based simulation scenario for telesimulation by using the virtual meeting platform Zoom® to meet our curricular needs. The use of telesimulation to teach a complex case-based scenario is novel. Two cohorts of anesthesiology residents participated 2 weeks apart. All learners were located at home. Four faculty members conducted the telesimulation from different locations within our simulation center in the roles of director, simulation operator, confederate anesthesiologist, and confederate surgeon. The anesthesiologist performed tasks as directed by learners. The scenario was divided into four scenes to permit reflection on interventions/actions by the participants based on the clinical events as the scenario progressed, to facilitate intermittent debriefing and learner engagement. All residents were given a medical knowledge pretest before the telesimulation and a posttest and learner satisfaction survey at the conclusion. The scenario was authentic and immersive, represented an actual case, and provided the opportunity to practice lessons that could be applied in the clinical setting. Participants rated telesimulation a reasonable substitution for in-person learning and expressed gratitude for continuation of their simulation-based education in this format during the pandemic. Participants in the second cohort reported feeling more engaged (*p* = 0.008) and stimulated to think critically (*p* = 0.003). Audio quality was the most frequently noted limitation. Fifty-three residents completed both pre- and posttests. The two cohorts did not differ in knowledge pretest scores (62% vs 60%, *p* = 0.80) or posttest scores (78% vs. 77%, *p* = 0.87). Overall, knowledge scores improved with the telesimulation intervention (pretest mean = 61% [SD = 14%]; posttest mean = 78% [SD = 12%]; *t* (41) = − 7.89, *p* < 0.001). Thus, using a Zoom format, we demonstrated the feasibility of adapting a complex case for telesimulation and effective knowledge gain. Furthermore, we improved our process in real time based on participant feedback. Participants were satisfied with their learning experience, suggesting that this format may be used in other distance learning situations.

## Introduction

The COVID-19 pandemic from SARS-CoV-2 virus has had a marked impact on medical education [1]. Anesthesiology residency has traditionally followed an apprentice model wherein most training takes place in the operating room (OR). However, postponement of elective surgery during the pandemic, as recommended by the American College of Surgeons, severely curtailed intraoperative learning opportunities [2, 3]. Furthermore, all in-person educational activities, including simulation, were halted at our institution. With no clear endpoint in sight, we, like others in the medical education community, were required to adapt our existing simulation curriculum for distance learning [4, 5].

Pandemic-mandated distance learning is relevant to all disciplines but is of particular concern for training anesthesiology residents. Lecture-based portions of our curriculum were easily transitioned to distance learning. However, teaching technical expertise, critical thinking, and decision-making in a time-sensitive context, as well as nontechnical skills such as communication, leadership, and situational awareness, are critical for competence in anesthesia [6] and represent an opportunity for innovation in distance learning. Conventional hands-on, simulation-based training has become an integral part of medical education during which trainees can practice technical and nontechnical skills in a safe learning environment with no risk to patients [7]. We were challenged to modify instructional techniques that best engage our learners, while also protecting their physical and mental safety. As many residents were redeployed to various clinical support roles in the intensive care units, we felt it necessary to supplement their experience with simulated intraoperative cases. We began by adapting an existing complex case-based simulation scenario, with which we have extensive experience, into a telesimulation learning experience.

Telesimulation was defined by McCoy et al. [8] as a distance learning method in which telecommunication and simulation resources are utilized together to build knowledge and provide skills training. It can also be used as a mechanism to assess offsite learners. Although this approach is still nascent, existing reports describe a combination of either remote control of a manikin and observation or remote observation paired with teledebriefing [9, 10]. Use of telemedicine training has been described for laparoscopic surgery, placement of intraosseous lines, and performance of regional anesthesia, but the focus has been predominantly on technical skills [11–13]. While telesimulation is effective for some task training, it is unclear if it would be an effective modality to enhance (improve) knowledge among anesthesiology trainees to manage a complex, case-based scenario, and how the residents would perceive this modality of education compared to our conventional in-person simulation.

Our conventional simulation curriculum employs scenarios with multiple time-sensitive, life-threatening events in an immersive OR environment. We believe it may be considerably more difficult to communicate physiologic urgency when learners are separated from authentic surroundings and lack visual, audio, and tactile cues.

The objectives of this study were:

1. Primary objective:
  - To evaluate whether it is feasible to reproduce a complex case-based scenario using telesimulation
  - To evaluate whether telesimulation (using Zoom-based web-platform) is a viable educational alternative to conventional in-person learning
2. Secondary objective:
  - To assess the effectiveness of telesimulation in education in training

## Methods

In this study, we evaluated the feasibility of telesimulation to reproduce a complex case-based scenario, from our existing immersive high-fidelity simulation curriculum for anesthesiology residents. We used pre- and posttest knowledge questions to evaluate the effectiveness of this method as a teaching technique and surveyed participants regarding the value and quality of the telesimulation experience. IRB Approval was obtained from the Johns Hopkins Institutional Review Board (IRB00241044). The details of the telesimulation procedures are described below.

### Existing simulation curriculum

Our institution is an academic tertiary-care hospital in the northeastern USA that trains approximately 85 anesthesiology residents annually. Residents have twice-monthly dedicated academic time, which includes 1 to 2 h of monthly simulation education per resident. Simulation education is typically conducted in person in our simulation center, where simulated ORs are outfitted with surgical and anesthesia equipment like that in our patient ORs. Laerdal (Wappingers Falls, NY) adult (SimMan 3G) and pediatric (SimBaby) manikins with full physiologic functioning are utilized. In most situations, we have two simultaneous simulated ORs with identical scenarios, each staffed by one faculty operator and one faculty confederate to facilitate smaller learning groups. Our simulation education faculty consists of practicing anesthesiologists who have experience as simulation operators, confederates, and debriefers. Simulation operators manipulate manikin responses from a concealed adjacent control booth. Our learners include clinical anesthesia first-, second-, and third-year residents (CA-1, CA-2, and CA-3, respectively). They are typically organized in 3–5-person groups, with varied representation from each year of training, depending on learning needs for the scenario.

Scenarios are often derived from real-life situations with a basis in experiential learning theory [14]. Residents reflect on their experience and actions in facilitated debriefing sessions, which enable development of concepts and abstractions that they can apply or test during another simulated or real patient encounter.

### Telesimulation scenario

We adapted an existing scenario in our simulation curriculum that was based on a real clinical case, retaining the original teaching points. An otherwise healthy 15-month-old child presents to the OR emergently to undergo cranial decompression for an intracranial epidural hematoma caused by a motor vehicle accident. The scenario starts with the patient on the OR table. The airway has been secured, mechanical ventilation has been established, and a single peripheral intravenous line and arterial blood pressure monitoring catheter are in place. Surgery is complicated by an unanticipated sagittal sinus injury. Consequently, massive hemorrhage leads to hemodynamic instability that requires resuscitation with blood transfusion. The resulting metabolic derangements are severe enough to cause cardiac arrest that requires implementation of Pediatric Advanced Life Support (PALS) protocols. The scenario resolves with the successful implementation of PALS and the recognition and correction of metabolic abnormalities.

The teaching points of the scenario are (1) anticipation of bleeding risk in pediatric epidural/subdural hematoma, (2) discussion of anesthetic management for traumatic brain injury and increased intracranial pressure, (3) identification of the causes of cardiovascular instability, and (4) recognition of pre-arrest physiology and successful implementation of PALS. To engage all of our off-site learners and address key teaching points, we paused the scenario at four key intervals to provide real-time feedback and coaching, or synchronous learner-instructor interactions.

### Telesimulation procedure

**Fig. 1:**
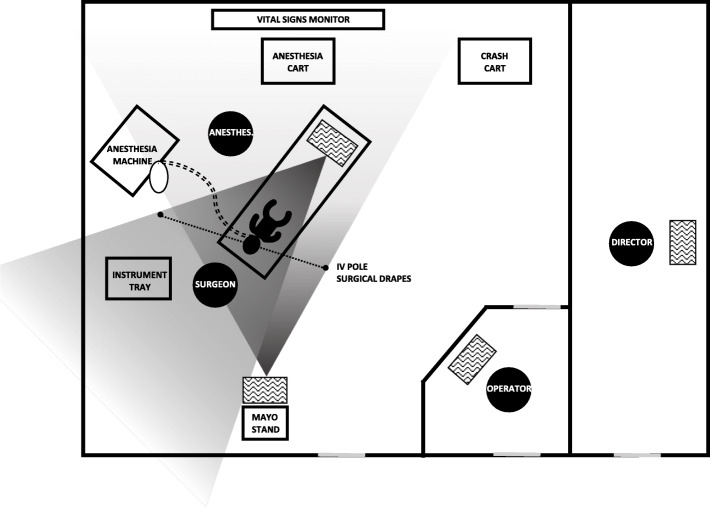
Simulated operating room. Schematic of simulated operating room layout with faculty (black circles) and laptop (rectangles with wavy lines) locations. Shaded cones represent approximate camera angles for video feed showing events in the simulated operating room to remote participants

Participants were scheduled to participate remotely in telesimulation via 1-h open Zoom (Zoom Video Communications, Inc., San Jose, CA) meetings. For each session, 6–8 residents participated in a group. A total of eight sessions were conducted on 2 days separated by 2 weeks (April 23, 2020, and May 7, 2020). Four faculty anesthesiologists were present in the simulated OR suite with a SimBaby manikin. Four portable laptop computers were used with the following simulation (and Zoom) roles: operator (host), director (co-host), confederate surgeon (co-host), and confederate anesthesiologist (participant). Table 1 shows the video and audio input and output for the computers of each faculty member and resident. Figure 1 shows the simulated OR layout with faculty and laptop locations.

**Table 1:** Video and audio inputs for resident and faculty computers during telesimulation procedure

Characteristic	Description
**Residents 1–8**	
Zoom role	Participant
Location	Home or office
Video received	Figure 2a, b: Picture-in-picture • Large: Spotlight video from confederate surgeon • Small: Share screen from operator (Laerdal patient monitor) or director (PowerPoint) Figure 2c, d: Side-by-side (If learner prefers to see video of peer learner participants) • Large: Spotlight video of OR with video of participant learners (gallery view) • Small: Share screen from operator (Laerdal patient monitor) or director (PowerPoint)
Audio received	Laerdal patient monitor; audio from OR via confederate surgeon; non-muted participants
Video output	Learner’s video
Audio output	Learner’s audio
**Simulation operator**	
Zoom role	Host
Location	Simulation control booth
Video received	Figure 3a: LLEAP software—operator control
Audio received	Figure 3a: LLEAP software on laptop; audio from OR to control booth via existing microphone system
Video output	Figure 3b: Laerdal patient monitor application via share screen; coordinates share screen with director
Audio output	Figure 3b: Laerdal patient monitor application via share screen; coordinates share screen with director
**Simulation director**	
Zoom role	Co-host
Location	Remote office
Video received	Figure 3c: Side-by-side • Large: Video of participant learners (gallery view) • Small: Share screen from operator (Laerdal patient monitor) or director (PowerPoint)
Audio received	Figure 3c: Non-muted participants including confederate surgeon and anesthesiologist, Laerdal patient monitor
Video output	Figure 3d: PowerPoint via share screen with scenario stem, QR codes for pre- and post-tests and evaluation; coordinates share screen with operator
Audio output	Figure 3d: Microphone on during briefing/debriefing; MUTED during scenario action
**Confederate surgeon**	
Zoom role	Co-host
Location	Simulated OR; head of OR table
Video received	Figure 4a: Side-by-side view • Large: Video of participant learners (gallery view) • Small: Share screen from operator (Laerdal patient monitor) or director (PowerPoint)
Audio received	Figure 4a: Non-muted participants; Laerdal patient monitor
Video output	Figure 4b: Overview of OR via spotlight video
Audio output	Figure 4b: Audio from OR confederates (surgeon, anesthesiologist)
**Confederate anesthesiologist**	
Zoom role	Participant
Location	Simulated OR; foot of OR table
Video received	Figure 4c: Side-by-side • Large: Video of participant learners (gallery view) • Small: Share screen from operator (Laerdal Patient Monitor) or director (PowerPoint)
Audio received	Figure 4c: SOUND OFF to prevent feedback
Video output	Figure 4d: 2nd view of OR from foot of the OR table
Audio output	Figure 4d: MUTED to prevent feedback

*OR* operating room, *QR* quick response

At the beginning of each session, participants joined the Zoom waiting room at the assigned time. To minimize interruptions to the learning experience, the director admitted them to the simulation session only after all scheduled participants were present. The director used the Zoom “share screen” option to display a Quick Response (QR) code for participants to complete a pre-scenario assessment of medical knowledge (via Qualtrics [Provo, UT]). Once the pre-test was complete, the director shared the scenario stem as described in Supplement S1.

### Zoom settings during simulation

**Fig. 2:**
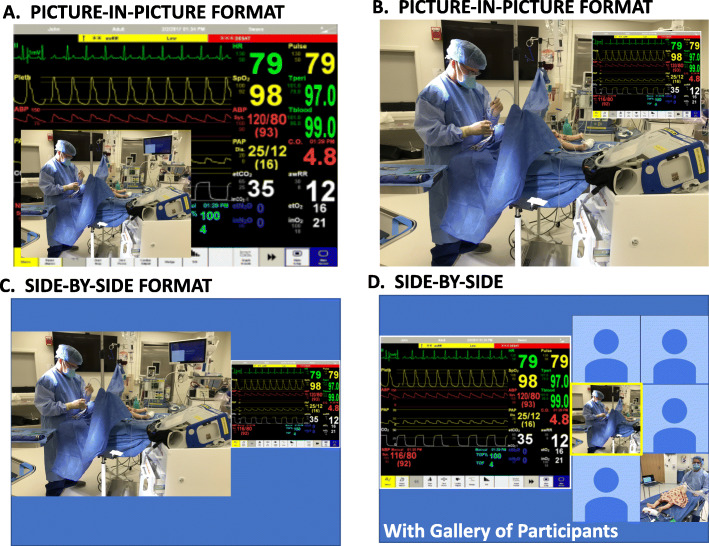
Participant’s view. Zoom screen views for the participants are customizable. Participants could view the “share screen” and the “spotlight video” in the default picture-in-picture format or choose side-by-side from the dropdown menu of “view options.” **a**, **b** Examples of the picture-in-picture format illustrate the operator’s shared screen of patient monitor vitals and the confederate surgeon’s “spotlight video” of the simulated operating room. **c** An example of side-by-side format with the same images highlighted. **d** Side-by-side format showing the gallery of participants and the view of the operating room from the confederate anesthesiologist’s laptop

**Fig. 3:**
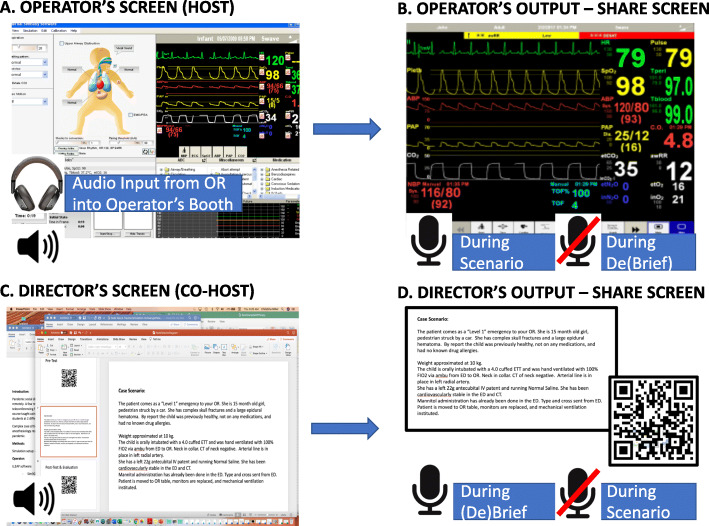
Operator’s and director’s views. Zoom screen views for the operator and director are customizable. **a** The operator’s laptop screen with the Laerdal operating platform (with audio) as well as audio from the simulated operating room (OR) delivered via headphones. **b** View of what the operator projects to participants, which includes audio heart tones from the patient monitor. The operator’s personal microphone is muted. **c** The director’s laptop screen with scenario stem or QR code as well as audio to hear all faculty and learner participants. **d** View of what the director projects to participants. The director has the option to use the microphone to speak directly to participants or mute during the scenario

**Fig. 4:**
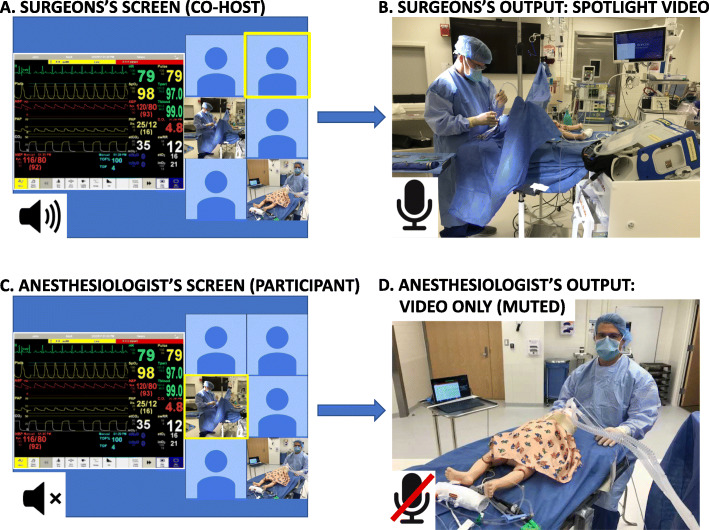
Confederate surgeon’s and anesthesiologist’s views. Zoom screen views for the confederate surgeon and confederate anesthesiologist are customizable. **a** The surgeon’s laptop screen with patient monitor and gallery of participants in side-by-side format and audio from the participants. **b** View of what the surgeon projects to participants with audio of activity in the simulated operating room. **c** The anesthesiologist’s laptop screen shows the patient monitor and the gallery of participants. Volume is silenced to prevent feedback (audio of participants is heard from the surgeon’s laptop). **d** View of what the anesthesiologist projects to participants (alternate view of the simulated operating room). Because the microphone is muted to prevent feedback, the anesthesiologist speaks into surgeon’s laptop, which has active audio

Using Zoom’s share screen feature, the operator projected the Laerdal patient monitor vital signs screen. The confederate surgeon used the Zoom command “Spotlight” video from within the simulated OR, which showed the manikin and overview of the OR. The director provided step-wise instructions for participants to optimize their view in Zoom, and participants controlled their own Zoom layout. For instance, if they used the picture-in-picture format (Fig. 2a, b), they saw two critical images, the vital signs monitor and the surgeon’s video overview of the OR. If they chose side-by-side format (Fig. 2c, d), they could see an alternate view of the OR or the gallery of participants. OR confederates and participants performed a sound check before the session was begun. Figures 3 and 4 illustrate the Zoom screen views for the operator (Fig. 3a, b), director (Fig. 3c, d), confederate surgeon (Fig. 4a, b), and confederate anesthesiologist (Fig. 4c, d).

### Participant roles

The participants were asked to respond to the scenario and verbalize all the actions that they would carry out in a real-life situation. The confederate anesthesiologist performed actions based only on directions from the resident participants and used closed-loop communication to indicate when an intervention they directed was complete. The confederate surgeon provided details of the surgery and spoke directly with participants. The operator reacted to all voiced instructions by controlling the simulated vital signs “on-the-fly” to indicate the patient’s physiologic responses to the interventions ordered by the residents and performed by the confederate surgeon and anesthesiologist. The director orchestrated intermittent pauses and facilitated reflection on the team’s management of the case, addressed the educational content, engaged participants who were not actively participating, and addressed any technical difficulties that arose.

### Reflection

The scenario was divided into four separate scenes to permit intermittent reflective pauses. Pauses were scheduled at targeted teaching moments that varied slightly based on the participants’ responses to events as they progressed. The four teaching moments identified were induction of anesthesia and preparation for anticipated severe blood loss prior to incision, intraoperative management of increased intracranial pressure and cerebral perfusion pressure, management of sudden blood loss resulting from injury to the venous sinus, and hyperkalemic arrest and successful implementation of PALS. We used the intermittent reflective pauses to permit periodic summary of interventions performed, allow participants to modify their plan based on the feedback, and assess how the modified simulation format was being received. At the end of the scenario, the director displayed a second QR code for participants to complete a posttest and learner satisfaction survey.

### Assessment

A total of 18 pre- and posttest knowledge questions were created (Supplement S1). These included questions relevant to the case as well as decoy questions intended to prevent clueing residents on the subject of the simulation. The posttest also included survey questions to elicit the value and quality of the telesimulation experience for the residents. A free-text item on the survey requested feedback for improving the telesimulation experience. We conducted item analysis for the test questions and discarded two questions based on poor item difficulty and/or discrimination. The final posttest with survey is included in Supplement S1, Additional file 1.

### Statistical analysis

For the knowledge test analysis, we calculated percentage scores by dividing each participant’s total number of correct answers by the total number of test questions. These percentage scores were used in the analyses and reporting. An independent samples *t* test was used to compare knowledge test scores of the two telesimulation sessions held 2 weeks apart, and a paired-samples *t* test was used to examine knowledge gain from pretest to posttest for all participants combined.

For Likert-style responses, assumptions for parametric tests were not met, as the Shapiro-Wilk’s test was significant. Therefore, we compared such responses using the nonparametric Mann-Whitney *U* test. Negatively worded items were reversed before the analyses and reporting. All statistical analyses were conducted with Statistical Package for the Social Sciences (IBM SPSS Statistics for Mac, Version 25.0. Armonk, NY: IBM Corp.), with significance level set at *p* < 0.05. We used thematic analysis to organize the free-text comments around common themes.

Based on initial feedback provided by the residents who participated in the telesimulation sessions on April 23, we made some adjustments to improve the educational experience for the sessions on May 7. Adjustments included optimization of sound quality and camera angles, and assigning leadership roles to specific senior residents.

### Handling missing data

Of the 58 residents who participated in the eight telesimulation sessions, 53 completed pretest and/or posttest measures. However, some data were missing. Only 42 residents completed both tests. Four residents did not complete the pretest and seven did not respond to the posttest. To make the best use of all responses, we did not use list-wise deletion for the posttest survey (*n* = 46). However, we did use a list-wise deletion for the knowledge test data, as it was required by the use of paired-samples *t* test.

## Results

Of the 53 respondents (27 female), 12 were CA1, 21 were CA2, and 20 were CA3. Results showed no difference in knowledge test scores between the two separate weeks on the pretest (62% vs 60%, *p* = 0.80) or posttest (78% vs. 77%, *p* = 0.87). All participants combined showed a statistically significant knowledge gain (pretest mean = 61% [SD = 14%]; posttest mean = 78% [SD = 12%]; *t* (41) = − 7.89, *p* < 0.001).

Overall, residents rated the learning experience positively. Residents felt that telesimulation could be a reasonable substitute for in-person learning in the simulation center (median = 7 on a scale of 0 [strongly disagree] to 10 [strongly agree]) and expressed gratitude for continuation of their simulation-based education in this format during the pandemic. The scenario was authentic and immersive, represented an actual case, and provided the opportunity to practice lessons that could be applied in the clinical setting. The lowest rated items (difficulty understanding the clinical flow of the case, feeling distracted by technology, ability to hear the facilitator and other participants clearly) were related to the technical challenges in conducting simulation remotely. We noted some significant differences between the two telesimulation cohorts based on the survey questions. The second cohort gave higher scores for engagement, reported being more stimulated in critical thinking and reflection during the session, and thought that the telesimulation was a reasonable substitute for in-person learning (Table 2).

**Table 2:** Comparison of results for posttest survey responses by week

Survey item	April 23 (***N*** = 27)		May 7 (***N*** = 19)		Overall (***N*** = 46)		***P***
	Mean (SD)	Median	Mean (SD)	Median	Mean (SD)	Median	
I thought the scenario was a realistic representation of a real-life situation	6.74 (2.77)	8	8.05 (2.20)	8	7.28 (2.61)	8	0.088
I felt engaged during the simulation	6.15 (2.82)	7	8.16 (2.5)	9	6.98 (2.85)	8	0.008
This simulation session stimulated critical thinking	7.93 (1.86)	9	9.11 (1.94)	10	8.41 (1.96)	9	0.003
I felt at ease in speaking up using this mode of simulation	5.52 (2.98)	5	7 (2.94)	8	6.13 (3.02)	7	0.098
The simulation case scenario was challenging	8 (1.39)	8	8.89 (1.24)	9	8.37 (1.39)	8.5	0.030
I had difficulty understanding the clinical flow of the case*	5.74 (2.98)	7	5.32 (3.53)	5	5.57 (3.19)	5.5	0.762
The principles I learned in this scenario can be applied in a real OR setting	9 (1.36)	10	9.53 (0.90)	10	9.22 (1.21)	10	0.171
The facilitation and debriefing allowed adequate reflection and learning	7.85 (2.23)	8	9.11 (1.29)	10	8.37 (1.98)	9	0.044
I could see the simulation room and activities clearly	6.19 (2.53)	6	7.11 (2.66)	7	6.57 (2.60)	7	0.180
I could hear the facilitator and other participants clearly	5.22 (3.14)	6	6.63 (2.89)	7	5.8 (3.09)	6.5	0.123
I felt distracted by technology or things going on in my viewing room*	5.22 (3.38)	4	6.47 (3.34)	8	5.74 (3.38)	5	0.274
Compared to learning live in the simulation center, this was a reasonable substitution	5.63 (2.96)	6	7.58 (3.36)	9	6.43 (3.24)	7	0.024

*p* values are based on Mann-Whitney *U* test. The scale is from strongly disagree = 0 to strongly agree = 10

*Indicates an item that was reversed so that a greater value reflects a more positive response

*OR* operating room, *SD* standard deviation

Additional information was obtained from free-text responses to the statement “Please share any ideas you have for improving the experience.” Formal qualitative analysis was not pursued, though we did find it useful for creating some common themes and representative quotes (Table 3).

**Table 3:** Themes identified in free-text responses to the survey statement “Please share any ideas you have for improving the experience”

Themes	No. (%) of related comments	Representative quotes
Better noise control and improve hearing	14 (38)	- There were some issues with background noise that could be improved upon for next time - … position a camera above the operating bed and make actors wear mics - It would be helpful to decrease the volume of the monitors.
Not the same as in-person but (with some adjustments) a viable option to continue sim-based education	13 (35)	- Loved this virtual sim! Fun, really challenged our clinical reasoning skills, and flowed well! - I think given the circumstances, this was a valuable learning experience - The sim was surprisingly way better than expected, considering all the limitations and the zoom overload.
Learner preparedness and team work	7 (19)	- Would be better in smaller groups like real sim. - I think all participants should be strongly encouraged to use video to simulate teamwork and a group environment. If it all possible we should be able to have an email or another screen with the case displayed for access during the SIM
Intermittent debriefing is effective for reflection	3 (8)	- I very much appreciated the stepped debrief sessions, which allow us to reflect on what we were thinking and what we could have done differently.
Total	37 (100)	– –

## Discussion

Our goal was to determine the feasibility and effectiveness of providing resident education via telesimulation using Zoom as a web-based interface. We implemented this substitute for the high-fidelity, immersive, hands-on simulation that is part of our normal curriculum to comply with social distancing requirements during the COVID-19 pandemic. We adapted a previously used scenario of a complex case that we knew could achieve our learning goals and objectives through hands-on simulation. Based on reports from others [4, 9–13], we thought that translating this scenario to telesimulation would be possible.

### Use of telesimulation to meet educational needs

Telesimulation permits dissemination of information and educational content to remote locations and collaboration among different centers or institutions [15, 16]. In addition, telesimulation saves time and travel costs. Telesimulation learners should reap many of the benefits of conventional simulation techniques such as engagement in real-life situations, synchronous learner-instructor interaction, feedback and real-time coaching, team communication practice, and the ability to reflect on one’s actions during facilitated debriefing. Studies demonstrating that telesimulation provides these benefits are promising [4, 5, 8]. In the current study, we describe the feasibility of telesimulation to simulate a complex case scenario. The participants responded that telesimulation was a reasonable option to conventional in-person simulation and lessons learnt from this session can be applied to real life situations.

### Novel application of Zoom (a web-based interface) for telesimulation

The use of Zoom to simulate a complex case scenario is novel, particularly with learners each at separate locations and instructors using multiple computers. We had to overcome the challenges of providing the necessary audio and video inputs and allowing residents to execute complex decisions through a live proxy. We tested several modes of Zoom capture to optimize the fidelity necessary to communicate the events of a crisis and intervene in real time. In addition, the learners must have access to devices with sufficient processing power, audiovisual capabilities, and Internet connectivity to support teleconferencing with high fidelity in real time.

### Challenges with telesimulation

- *Audio*

Audio representation presented one of the greatest challenges, as noise pollution from side conversations, echoes, and microphone feedback interfered with the voices of confederates and participants. Laptops in the simulation center needed to be distanced from each other or muted to prevent feedback (Fig. 1). Heart tones from the patient monitor, which are critical to indicating the patient status, competed with the confederates’ conversations that communicated the flow of the case. This interference was compounded by heart tones audible in the simulated OR, which were rebroadcast by the surgeon’s computer. The interference might be improved by diminishing the volume of the heart tones and allowing confederates to hear them through Zoom audio with earbuds to maintain better acoustic sterility in the OR. During this time, resources critical to telesimulation such as cameras and microphones were in high demand, and materials for optimizing video and audio quality were not always available. Additionally, muting the surgeon’s computer, rather than the anesthesiologist’s, may have permitted better sound quality communication between participants and their direct proxy.

b) *Optimizing video*

Another challenge was optimizing video input with the use of Zoom features to create a dual screen experience of patient vital signs broadcast by the operator and live video from the simulated OR. It was essential for the director to ensure that all participants configured their screens before the simulation began in a way that would enable them to view the critical elements and hear confederates and fellow participant team members.

Future telesimulation efforts may benefit from the use hardware or software that permits the director to integrate multiple video and audio inputs into a consistent production feed without the need for individuals to configure their own display.

c) *Learner engagement during telesimulation*

We were also faced with the question of how to engage all of the residents in decision-making and elicit the rationales and critical thought processes that drove their decisions. Whereas the confederate surgeon had scripted motivation and would continue operating without direction, the anesthesiologist acted as a proxy with no inherent motivation and waited for verbal direction from participants who could not physically intervene. In some groups, residents were hesitant to take control and speak up. Distance participation may encourage learners to try to remain anonymous. Some residents chose to use the “chat” function in Zoom rather than to speak. It was difficult for the director to follow this mode of communication because of competing tasks. In the future, use of the chat function should be discouraged or monitored by an additional person.

Assessing learner engagement was often difficult during telesimulation. Engagement is defined as emotional, behavioral, and cognitive involvement in academic activities for the purpose of learning, problem solving, and mastering skills [17]. Cognitive involvement might be approximated by improvement in knowledge scores. However, removal of environmental context and physical interaction between team members dampened non-verbal social cues, and the small Zoom gallery made it difficult for faculty to see behavioral or emotional involvement. Furthermore, some learners participated without enabling their own video or muted themselves periodically throughout the activity, making it more difficult to assess their level of engagement.

Larger participant groups of 6–8 residents (compared to our typical groups of 3–5) may have contributed to learners feeling less engaged. We needed to use larger group sizes because personnel resources were limited, forcing us to conduct fewer overall simulations. Based on our experiences, we encourage others to keep group sizes as small as possible, assign roles, and use scenario pauses for intermittent reflective pauses.

### Reflective pauses during telesimulation

Given the importance of debriefing to simulation learning [18], the director included pauses in the scenario for targeted Socratic teaching and to ensure that no resident was lost in the action. These breaks provided opportunities for learners to pause and reflect on their current actions and thought processes, modify their existing diagnoses and action plans, and apply the newly acquired information to the ongoing scenario. The educational framework utilized during our telesimulation is congruent with reflection-in-action as described by Donald Schon, allowing the learner to stop, think, re-evaluate, and reshape while doing a task [19]. These intermittent reflective pauses also permitted faculty to directly engage individual learners and assess their level of comprehension and may have helped less-engaged residents derive more benefit from the experience. Based on this experience during the early groups, the director assigned leadership roles to specific senior residents in subsequent groups. Having an assigned team leader(s) made it easier to maintain the flow of verbal directions to the confederate anesthesiologist and maintain a desired pace of the simulated case. In addition, the pre-test primed the residents to seek relevant learning material during the simulation.

Conducting simulation sessions 2 weeks apart permitted us to learn from experience and make modifications to create a better experience for the second cohort. Our results suggest that participants found the educational utility of the telesimulation to be satisfactory, if not high. Interestingly, there were no significant differences in results related to technical difficulties.

Although this format was designed out of necessity during the COVID-19 pandemic, we see applications that reach beyond mandated social distancing. For instance, telesimulation offers opportunities to present educational materials to learners who are geographically distant or do not have access to physical simulation resources. Additionally, it can be used to facilitate multicenter educational activities or to enhance generalizability regarding educational research interventions.

## Limitations

Limitations of this study include its small sample size. The rapidly evolving pandemic mandated that we develop increased facility with telesimulation in a short period of time or risk cancelling previously scheduled educational activities. We developed this modified program with very little advanced notice, learning as we went. We did not collect data on previous iterations of this scenario that could afford direct comparison of in-person and telesimulation techniques. As a result, we relied on a pre- and posttest method to demonstrate that learning had taken place and asked participants to compare this event to their past experience with different in-person scenarios. One final drawback was that telesimulation learners relied on a live proxy to physically perform their interventions and thus were unable to create “muscle memory” with this method.

## Conclusion

The use of telesimulation for high-fidelity, immersive, case-based simulation using Zoom technology is feasible and a reasonable alternative to conventional in-person simulation. It is effective at increasing knowledge of subject matter, and participants were satisfied with their learning experience. Our findings suggest that this format may be used in other distance learning situations for locales that do not have access to physical simulation resources, or to facilitate multicenter educational activities and enhance generalizability regarding educational research interventions. We hope that our experience encourages others to think creatively about individualizing learning when adaptation is necessary and to use telesimulation in additional, novel ways.

## Supplementary information

**Additional file 1.** Case description and survey questions.

## Data Availability

The de-identified datasets used and/or analyzed during this study are available from the corresponding author on reasonable request.

## Abbreviations

OR: Operating room
CA-1: Clinical anesthesia first-year residents
CA-2: Clinical anesthesia second-year residents
CA-3: Clinical anesthesia third-year residents
PALS: Pediatric Advanced Life Support
QR: Quick response
SD: Standard deviation
