# Supplementary material for: The sim must go on: adapting resident education to the COVID-19 pandemic using telesimulation
Source: Adv Simul (Lond). 2020 Sep 29;5:26. doi: 10.1186/s41077-020-00146-w (PMC7522907; doi:10.1186/s41077-020-00146-w)
Supplement: Supplementary file 1 — Additional file 1. Case description and survey questions. [file 41077_2020_146_MOESM1_ESM.docx]

**Supplement S1**

Scenario Stem

An otherwise healthy 15-month-old presents to the operating room emergently to undergo cranial decompression for an intracranial epidural hematoma sustained in a motor vehicle accident. The patient presents with a secured airway, single peripheral intravenous access, and arterial catheter in place. The scenario starts with patient on the operating room table, monitors attached, and mechanical ventilation established.

Demographic questions:

1. I am a:
2. CA3
3. CA 2
4. CA 1
5. Medical Student
6. My unique identifier is (please use the same number on pre and post-test, suggest last 4 digits of social security or phone).

Knowledge questions:

1. What is the estimated blood volume in a term neonate?
2. 90 ml/kg
3. 80 ml/kg
4. 100 ml/kg
5. 75 ml/kg
6. Which of the following is an indicator of improper CPR in a 1-year-old?
7. Rate of 100-120/min
8. Depth 2-2.5 inches
9. Diastolic BP > 25 mmHg
10. In case of a cardiac arrest in a pediatric patient, what is the correct dose of epinephrine?
11. Epinephrine 0.01 mcg/kg IV
12. Epinephrine 0.1 mg/kg IO
13. Epinephrine 0.01 mg/kg ETT
14. Epinephrine 10 mcg/kg IV
15. Sudden cardiac arrest associated with MH is a result of:
16. Massive breakdown of muscle tissue
17. Direct cardiotoxicity from exposure to halogenated volatile anesthetics
18. Rapid reuptake of calcium into sarcoplasmic reticulum
19. The use of calcium channel blocker to treat dysrhythmias during MH is contraindicated.
20. True
21. False
22. Anemia is present in over 90% of infants with epidural hematoma.
23. True
24. False
25. After successful treatment of an intraoperative MH crisis, the patient should be monitored for at least 24 hours:
26. In the ICU, receiving dantrolene
27. On the floor with frequent vital signs
28. In the ICU with close observation and frequent blood draws

8. A ten year old with history of MVA presents to the ED disoriented and confused, opening his eyes spontaneously and flexion withdrawal to pain. What is his GCS score?

a) 15

b) 6

c) 12

d) 10

9. What are the structures shown in this picture? Write in your answer.


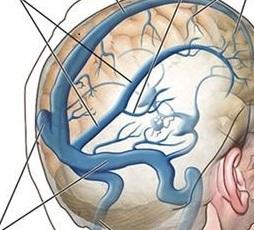


1. A 15-month-old with history of head injury following an MVA and GSC of 5 was emergently intubated in the ER with a 4.0 cuffed ETT secured at 14 cm at teeth. Patient is on 100% FiO_2_, PIP: 18, Rate 25/min, PEEP 4. The SaO_2_ decreases to high 80s. What do you think is the most likely cause?
2. Pneumothorax
3. Pulmonary contusion
4. Mainstem intubation
5. After 2 shocks and persistent V Fib, what should be your next step?
6. Administer epinephrine
7. Administer amiodarone
8. Administer lidocaine
9. Administer another shock
10. The most common presenting sign of epidural hematoma in infant is:
11. Loss of consciousness
12. Vomiting
13. Irritability
14. Bulging fontanel
15. Children with traumatic epidural hematomas have the best prognosis in which age group?
16. 0-2 years
17. 3-6 years
18. 7-15 years
19. The most sensitive sign suggestive of malignant hyperthermia:
20. Hyperthermia
21. Tachycardia
22. Rapid increase in end-tidal CO_2_ unresponsive to changes in minute ventilation
23. Intraoperatively there is sudden loss of end-tidal CO_2_ and diagnosis of cardiac arrest. The surgery resident starts CPR. What is the appropriate rate for a 15-month-old?
24. 80-100 compressions/min
25. 100-120 compressions/min
26. 120-150 compressions/min
27. The middle meningeal artery is the most common source of bleeding in children with traumatic epidural hematoma.
28. True
29. False
30. In case of ventricular fibrillation persisting after shock, the nurse asks you what dose of shock energy to charge for the second defibrillation (patient weight 10 kg)?
31. 10 J
32. 20 J
33. 50 J
34. Outcome of epidural hematoma is best predicted by which of the following at time of surgery?
35. Hemoglobin
36. GCS
37. Heart rate

Simulation Experience:

1. In the next section please think about the experience of learning using telesimulation based on a Likert scale (0-10, where 0 represents strongly disagree and 10 strongly agree):
2. I thought the scenario was a realistic representation of a real-life situation
3. I felt engaged during the simulation
4. This simulation session stimulated critical thinking
5. I felt at ease in speaking up using this mode of simulation
6. Please reflect on the learning experience of this particular case based on a Likert scale (0-10, where 0 represents strongly disagree and 10 strongly agree).
7. The simulation case scenario was challenging
8. I had difficulty understanding the clinical flow of the case
9. The principles I learned in this scenario can be applied in a real OR setting
10. The facilitation and debriefing allowed adequate reflection and learning
11. In this section please tell us about the operational experience for you, based on a Likert scale (0-10, where 0 represents strongly disagree and 10 strongly agree).
12. I could see the simulation room and activities clearly
13. I had difficulty understanding the clinical flow of the case
14. I could hear the facilitator and other participants clearly
15. I felt distracted by technology or things going on in my viewing room
16. Compared to learning live in the simulation center, this was a reasonable substitution
